# "Teaching: Individual" to increase adherence to therapeutic regimen in people with hypertension and type-2 diabetes: protocol of the controlled clinical trial ENURSIN.

**DOI:** 10.1186/s12912-019-0344-0

**Published:** 2019-06-04

**Authors:** Sandra Lucrecia Romero Guevara, Dora Inés Parra, Lyda Z. Rojas

**Affiliations:** Universidad Industrial de Santander - Escuela de Enfermería, Carrera 32 # 29-31 Facultad de Salud, Bucaramanga, Colombia

**Keywords:** Nursing Process, Diabetes Mellitus, Type 2, Hypertension, Patient Education as a Topic, Patient Compliance, Medication Adherence

## Abstract

**Background:**

Worldwide, hypertension affects approximately 25% of the adult population and diabetes about 8.5%. Lack of adherence to prescribed treatment regimen remains a problem among patients undergoing long-term treatment, showing high non-adherence rates, at estimated range of between 36 and 93%. In our city, patients with hypertension and diabetes in primary care are looked after mainly by doctors with little nursing support; also, there is no published dataset among Colombian populations on the effect of nursing intervention to increase adherence to therapeutic regimen. The aim of this study was to evaluate the efficacy of nursing intervention “Teaching: Individual” compared with usual care, to increase adherence to therapeutic regimen in people with hypertension and/or type-2 diabetes, and to analyze the impact to glycosylated hemoglobin and systolic blood pressure levels.

**Methods:**

A two-arm, single-blinded, randomized controlled trial, with participants allocated to either intervention group with “Teaching: Individual” provided by two nurses, or control group receiving routine care only. Two Hundred patients attending cardiovascular risk programs of Bucaramanga, Colombia were included. Nursing intervention consisted of six educational sessions about Coping Enhancement; Behavior Modification; Teaching: Disease Process, Prescribed Medication, Prescribed Diet and Prescribed Exercise. The outcomes were Treatment Behavior: Illness or Injury (adherence to treatment), levels of both glycosylated hemoglobin (HbA1c) and systolic blood pressure for 24 h, to be measured at baseline and two follow-up time points. Basic characteristics of the groups were compared through chi-square/Fisher’s exact or Students-T/Mann-Whitney U test. Outcomes were evaluated with repeated data methods and investigated changes in the outcomes over time and to compare these changes among treatment groups, and statistical significance with *p*-value < 0.05 were considered.

**Discussion:**

The nursing intervention “Teaching: Individual” to increase adherence to therapeutic regimen in people with hypertension and/or type-2 diabetes represents an innovative care approach intended for low-income population. The study will advise district health system policy makers and managers as to the efficacy of implementing this intervention. Should this intervention turn out efficacious, it can potentially achieve wide application in cardiovascular risk programs.

**Trial registration:**

ENURSIN was registered in ClinicalTrials.gov (NCT02758275) on April 27, 2016, protocol number 01.

## Background

Worldwide, hypertension affects approximately 25% of the adult population and about 346 million people with diabetes (80% of them live in low-and-middle-income countries) [1, 2]. The prevalence of these risk factors is increasing and will continue to grow as the population ages [3]. Then, in the future, complications of hypertension and diabetes can become the main threats to public health resources in the world, with huge economic and social cost [2, 4, 5].

The recommendations for treatment of hypertension and diabetes are pharmacological treatment and lifestyle modification: salt restriction, moderate alcohol consumption, high intake of fruits and vegetables, low-fat diet, weight reduction and regular physical activity. Adherence to these guidelines is an essential key to successful handling of these risk factors [6, 7]. However, lack of adherence to prescribed treatment regimen remains a problem among long-term treatment patients, who show high non-adherence rates, estimated to range between 36 and 93% [8].

A variety of strategies and techniques can be used to provide adequate education for the management of hypertension and diabetes [9]. Given that in our city, hypertension and diabetes patients in primary care are looked after mainly by doctors with little nursing support, we have designed a nursing intervention based on the results of a systematic review, where the authors found that the most effective interventions employ different components and deliver them along many days. Such components are: linking adherence behavior to habits, giving adherence feedback to patients, self-monitoring of blood pressure, using pill boxes and other special pill packaging, and motivational interviewing [10].

A nursing intervention is defined by *Nursing Intervention Classification* (NIC) as any treatment based on clinical knowledge and judgement conducted by nursing staff aimed at favoring those results expected of the patient [11]. Despite the importance of the previous, there is no published dataset among the Colombian population on the effect of nursing intervention to increase adherence to therapeutic regimen.

Then, our hypothesis is that the nursing intervention “Teaching: Individual” [11] is more effective to increase adherence to therapeutic regimen in people with hypertension and/or type-2 diabetes mellitus than usual care (control group), and it is better at decreasing levels of glycosylated hemoglobin and systolic blood pressure.

Consequently, the ENURSIN trial is designed as a randomized, controlled, parallel group, two-arm, double blinded comparing “Teaching: Individual” intervention [11] with usual care (Control group). The specific aims of the study are: 1) to evaluate the efficacy of nursing intervention “Teaching: Individual” [11] compared with usual care (control group), to increase adherence to therapeutic regimen in people with hypertension and/or type-2 diabetes mellitus and 2) to analyze the impact of nursing intervention to decrease systolic blood pressure and glycosylated hemoglobin levels in people with hypertension and diabetes.

## Methods

The clinical trial protocol is reported according to SPIRIT guidelines/methodology [12]. The study was registered at ClinicalTrials.gov under registration number NCT02758275 on April 27, 2016.

### Study design

This was a parallel group, two-arm, single-blinded, randomized controlled trial, comparing “Teaching: Individual” intervention [11] with usual care along 12-month follow-up.

### Setting

We recruited patients from cardiovascular risk programs of 21 primary care centers of Empresa Social del Estado Instituto de Salud de Bucaramanga (ESE-ISABU), Santander, Colombia. ESE-ISABU is a public health institution providing services with an emphasis on primary care, oriented towards user satisfaction and safety, and also with a teaching vocation.

### Inclusion and exclusion criteria

Criteria for patient inclusion: aged ≥18 years, medically diagnosed with hypertension and/or type-2 diabetes mellitus; active participant of primary care at cardiovascular risk programs of the ESE-ISABU, independent to conduct daily life activities, residents of Bucaramanga city and have access to landline or mobile telephone.

For exclusion, show either mental sphere changes according to Abbreviated Mental Test Minimental) [13] and Yesavage depression test [14], or communication limitations or chronic or serious alterations impairing intervention comprehension. Also, participation in a research study or having done so in the previous 6 months. Recruitment ran from April 2016 to June 2016.

### Sample size

**Fig. 1:**
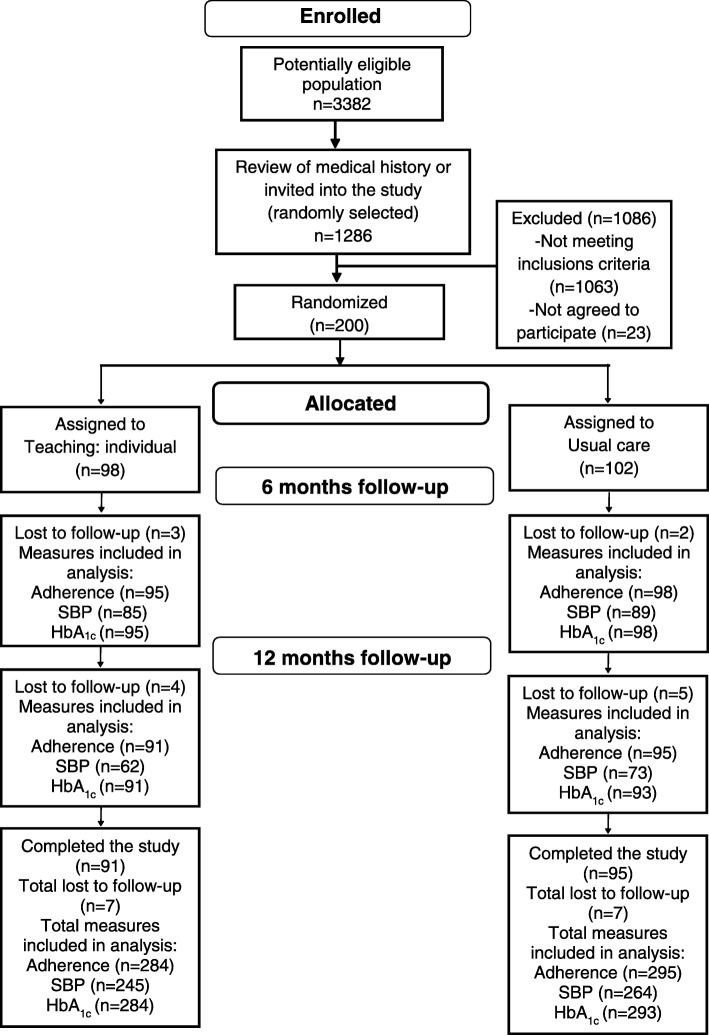
Consort flow diagram

Sample size was calculated taking into account the following parameters: an expected difference of 0.5 (SD 0.9) in the score results of the evaluation of the Treatment Behavior: Illness or Injury (adherence to treatment) between groups; 10 (SD 21) mmHg the difference in systolic blood pressure levels, a parameter that was determined in the investigators’ preliminary work and 0.5% (SD 1.2) difference in glycosylated hemoglobin levels [15, 16], with 80% power, 5% alpha, 0.3 average correlation between initial and final measuring, ratio intervened group/control group 1:1 and 20% adjustment due to follow-up losses, resulting in a sample size of 200 subjects (98 subjects for intervention group and 102 for control group) (Fig. 1).

### Randomization and blinding

All the study participants were randomized to be assigned to either the intervention group (Teaching: Individual and usual care) or control group (only usual care). The routine randomization for the study was developed as a function within an application hosted on the web servers of the Administration and Data Analysis Unit (UAAD) of CARDIECOL.

The randomization process had an algorithm that considered input parameters of each participant: informed consent, age, sex, diseases (hypertension, type 2 diabetes or both), as well as their identification code for stratification and, according to the layer, assigned them to either of the groups, keeping balance of the number of participants assigned to each group.

Access to the data in the platform was restricted according to the roles. Principal investigators (SLR and LZR) and UAAD had access to the cleaned data sets. To ensure confidentiality, data dispersed to project team members were blinded of any identifying participant’s information.

Only one of the researchers (SLR) had access to the platform for randomization of participants and was in charge of delivering intervention subject codes to intervention nurses.

While nurses who performed Teaching: Individual only had contact with intervention group, nurses who made the measurements, and person who performed the data analysis (LZR) were blinded to group assignment.

### Design of intervention

#### Intervention group (Teaching: Individual)

The intervention group received, in addition to the usual care, the Teaching: Individual Intervention [11], defined as “Implementation of the planning and evaluation of a teaching program designed to meet the particular needs of a patient” [11].

Patients were guided through the decision-making process and emphasis was placed on the motivational component; in each of the sessions the commitments and tasks of the previous session were evaluated, then the theme of the session was presented with the support of the educational material and the use of strategies and tools favoring the patient’s active participation (example: preparation of medication card, organization of pillboxes, elaboration of menus according to diet recommendations, tastes and economic resources, etc.). Finally, patient’s doubts and concerns were solved, and commitments and tasks related to the subject were established jointly by patient and nurse.

**Fig. 2:**
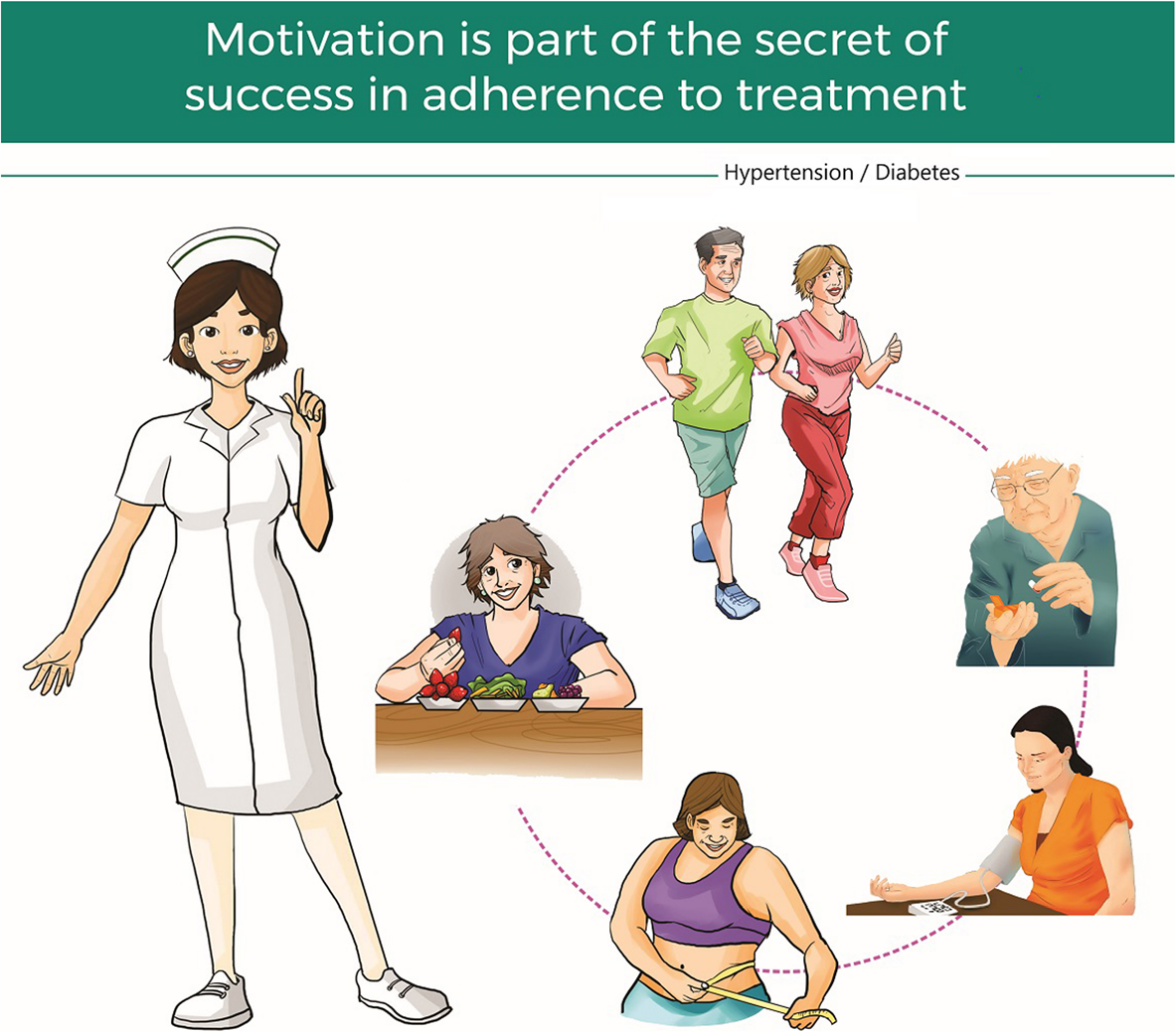
Picture of the booklet used for the Teaching: Individual. Source: authors

The intervention consisted of six educational sessions given by two trained nurses (three each), with topics and order shown in Table 1; session periodicity was monthly, lasting between 20 and 40 min each (Table 1). Participants received their own educational material (Fig. 2). Compliance with measurement and intervention procedures was audited by the researcher (DIP) through direct supervision and review of intervention application video. Intervention lasted from April 2016 to November 2016.

**Table 1:** Description sessions at the intervention group

Sessions [11]	Definition [11]	Duration (minutes)	Support material
Behavior modification	Promotion of a behavior change	20–30	−Educational booklet with worksheets: motivation is the key to successful compliance with my treatment. Hypertension Diabetes mellitus
Teaching: Disease Process	Assisting the patient to understand information related to a specific disease process	20–40	−Educational booklet with worksheets: once aware of my disease, I will assume my own care responsibly: hypertension and/or diabetes mellitus
Teaching: Prescribed Medication	Preparing a patient to safely take prescribed medications and monitor for their effects	20–40	−Educational booklet with worksheets: medication is the key to controlling hypertension and/or diabetes mellitus −Pillbox −Medicaments card
Teaching: Prescribed Diet	Preparing a patient to correctly follow a prescribed diet	20–30	−Educational booklet with worksheets: Feed yourself properly, and feel healthy: pick your own recipe for control of hypertension and/or diabetes mellitus
Teaching: Prescribed Exercise	Preparing a patient to achieve and/or maintain a prescribed level of activity	20–30	−Educational booklet with worksheets: I exercise my body, improve my health and help control my disease. Hypertension Diabetes mellitus
Coping Enhancement	Assisting a patient to adapt to perceived stressors, changes, or threats that interfere with meeting life demands and roles	20–30	−Educational booklet with worksheets: I control my stress and improve my physical and mental health

#### Control group

Participants continued receiving usual care in the health center they usually attended for medical controls. The usual care consisted of interdisciplinary management by the health team providing care, according to classification of the patient’s cardiovascular risk. Anamnesis, physical examination, prescribed medicament treatment, education and control of modifiable factors (diet, physical activity, alcohol, tobacco, adherence to treatment among others), laboratory and diagnosis tests were usually performed in medical controls (Table 2).

**Table 2:** Frequency of checks and laboratories according to the patient’s cardiovascular risk (usual care)

	Cardiovascular Risk			
Health professional	Low	Moderate	High	Very high
General physician	Six-monthly	Quarterly	–	–
Nurse	Six-monthly	Six-monthly	Annual	Annual
Nutritionist	Annual	Six-monthly	Six-monthly	Six-monthly
Psychologist	Annual	Six-monthly	Six-monthly	Six-monthly
Internal medicine physician	–	Annual	Four-monthly	Quarterly
Cardiologist	–	–	Annual	Annual
Nephrologist	–	–	Annual	Six-monthly
Neurologist	–	–	–	Annual
Ophthalmologist	–	–	–	Six-monthly
Medical test^a^	Low	Moderate	High	Very high
Hemogram	Six-monthly	Annual	Six-monthly	
Basal glycaemia	Annual	Annual	Quarterly	
Lipidic profile	Annual	Annual	Annual	
Partial urine	Annual	Annual	Annual	
Serum creatinine	Annual	Annual	Annual	
Electrocardiogram	Six-monthly	Six-monthly	Annual	
Glycosylated hemoglobin	–	–	Quarterly	
Microalbuminuria	–	Annual	Annual	
Echocardiogram	–	Six-monthly	Six-monthly	
Potassium	–	–	Annual	
Doppler	Initially	–	–	

Information provided by Empresa Social del Estado Instituto de Salud de Bucaramanga (ESE-ISABU). ^a^The medical tests are done upon admission to the program

### Outcomes

Primary outcome: change of mean adherence score from baseline (0 month) at 6 and 12 months, measured by Treatment Behavior: Illness or Injury Questionnaire [17]. Upon evaluation of the construct validity and reproducibility of this questionnaire, we found adequate performance of these aspects, supporting their utilization. The results of the instrument validation are currently in publication process.

Secondary outcomes: changes of mean score glycosylated hemoglobin levels from baseline (0 month) at 6 and 12 months, measured in peripheral venous blood, analyzed by immunoturbidimetry in whole blood by accredited laboratory, and changes of mean systolic blood pressure levels in 24 h from baseline at 6 and 12 months, measured by ambulatory blood pressure monitoring (WatchBP 03-microlife).

The measures that indicated risk to the integrity of the patient were reported in a timely manner to the professionals in charge of the care. The ethics committees along the study additionally conducted follow-up audits. Partial and final results of the study, as well as its technical aspects, were disclosed to the participants, the sponsors and the institutions as part of the researchers’ commitment to them.

With respect to baseline characteristics of study participants, no statistically significant differences were found between intervention group and control group (Table 3).

**Table 3:** Baseline characteristics of participants

Characteristics	Total (*n* = 200)	Study Group		*p*-value
		Teaching: Individual (*n* = 98)	Usual Care (*n* = 102)	
Sociodemographic				
Age (years)	62.8 ± 11.1	62.8 ± 11.6	62.7 ± 10.7	0.948
Sex				
Women	146 (73.00)	72 (73.47)	74 (72.55)	0.883
Men	54 (27.00)	26 (26.53)	28 (27.45)	
Marital status				
Married/living in a free union	98 (49.00)	50 (51.02)	48 (47.06)	0.950
Divorced	19 (9.50)	9 (9.18)	10 (9.80)	
Single	48 (24.00)	23 (23.47)	25 (24.51)	
Widowers	35 (17.50)	16 (16.33)	19 (18.63)	
Socioeconomic status				
Low	174 (87.00)	84 (85.71)	90 (88.24)	0.596
Medium	26 (13.00)	14 (14.29)	12 (11.76)	
Year of schooling	5 (2–5)	4 (2–5)	5 (2–5)	0.443
Occupation				
Unemployed	22 (11.00)	9 (9.18)	13 (12.75)	0.455
Employees	13 (6.50)	4 (4.08)	9 (8.82)	
Housewives	117 (58.50)	60 (61.22)	57 (55.88)	
Independent	47 (23.50)	24 (24.49)	23 (22.55)	
Pensioners	1 (0.50)	1 (1.02)	0 (0.00)	
Clinics				
Diseases				
Type 2 diabetes	24 (12.00)	11 (11.22)	13 (12.75)	0.944
Hypertension	125 (62.50)	62 (63.27)	63 (61.76)	
Hypertension/Type 2 diabetes	51 (25.50)	25 (25.51)	26 (25.49)	
Charlson index (points)	1 (0–1)	1 (0–1)	1 (0–1)	0.913
Tobacco consumption in the last year	12 (6.15)	6 (6.25)	6 (6.06)	0.956
Body mass index (kg/m^2^)	29.1 ± 5.4	29.5 ± 5.5	28.7 ± 5.3	0.349
Waist-hip index	0.89 ± 0.07	0.90 ± 0.07	0.89 ± 0.08	0.440
Cholesterol (mg/dl)				
Total	196 ± 40	200 ± 34	192 ± 45	0.165
Low density lipoprotein (LDL)	114 ± 37	118 ± 36	111 ± 38	0.248
High density lipoprotein (HDL)	48 ± 12	48 ± 12	48 ± 11	0.838
Triglycerides	173 ± 84	165 (115–204)	152 (116–207)	0.257
Outcomes				
Adherence score (points)	9.39 ± 2.05	9.39 ± 1.97	9.38 ± 2.13	0.957
SBP in 24 h (mmHg)	124 ± 14.3	125 ± 14.6	123 ± 13.9	0.385
HbA_1c_ (%)	6.18 ± 1.58	6.19 ± 1.71	6.15 ± 1.44	0.879

This table contains n (%) for categorical variables and mean (standard deviation) or median (first and third quartile) for continuous variables

Abbreviations: *SBP* Systolic blood pressure, ambulatory blood pressure monitoring; HbA_1c_ = Glycated haemoglobin

### Data collection

Clinical histories of potentially eligible patients were both selected through simple random sampling, and reviewed to verify inclusion criteria. Patients meeting the criteria underwent Abbreviated Mental (Minimental) [13] and Yesavage depression testing [14], to verify absence of mental sphere alterations or serious communication limitations.

People meeting all the criteria were invited by a study nurse to inform them about the research project, request their decision to participate and sign the informed consent.

Nurses applied questionnaires and took physical measurements, while laboratory assistant conducted blood testing. Baseline characteristics included age, gender, marital status, socioeconomic status, area of residence, schooling, occupation, pathology suffered, smoking status, weight, height, waist and hip circumference, abbreviated Charlson index.

Prescribed medication, HDL, LDL, total plasma cholesterol levels and plasma triglycerides levels were taken from medical records. Patients who did not wish to participate in the study were asked for consent to include their social characteristics, levels of adherence to treatment and reasons for not participating, to enable comparison with the participant group.

Direct phone contact was kept with participants to promote their retention and complete follow-up, reminding them of the educational session appointments and upcoming data collection; their transport to intervention and measurement site was funded by the project. All reports on glycosylated hemoglobin levels and ambulatory blood- pressure monitoring were delivered to the patients. We asked participants quitting the study the reason for such. Follow-up was from October 2016 to August 2017.

The investigators (SLR and LZR) and UAAD were given access to error-free data sets. To ensure confidentiality, data received by project team members were blinded of any information identifying participants. On the other hand, in order to guarantee data quality, the project’s epidemiologist (LZR) conducted periodic recorded data audits, according to the operating manual of the study.

### Statistical analysis

Analyses were based on intention to treat, where all participants remained in the allocated group irrespective of compliance with the protocol. Description of the categorical variables was made using absolute and relative values, and if quantitative values showed normal distribution for the Shapiro Wilk test, histograms and box plots, mean and standard deviation were reported; otherwise, the median and the first and third quartiles were reported. Baseline characteristics of the groups were compared through chi-square/Fisher’s exact or Students-T/Mann-Whitney U test, as suitable.

We have modified statistical analysis of outcomes approved in the protocol register (ClinicalTrials.gov), because it was basic analysis, and to compensate for the losses that occurred during the follow-up. To make use of repeated measure data and investigate changes in an outcome over time, and to compare these changes among treatment groups, we have used: linear marginal model with an exchangeable correlation matrix for repeated measures (SBP) and generalized estimating equations (GEE) for repeated measures with an unstructured correlation matrix (treatment adherence and HbA_1c_) using all available data [18, 19]. We used models predicting outcomes from treatment group, time, and the treatment group by time interaction. All analyses were conducted using Stata version 15.0; *p*-values < 0.05 were considered statistically significant.

### Bias control

The following measures were taken for bias control: (a) *selection bias:* so as to prevent this bias, selection of patients and intervention assignment were at random, *(b) information bias:* nurses evaluating the outcomes and the person conducting analysis were blinded to study group allocation. All procedures were standardized and supervised, and nurses were trained to assess outcome and deliver intervention. There was supervision, standardization and training of nurses in charge of evaluating the outcomes and conducting the intervention. Video-taped counselling sessions were constantly analyzed and fed back to maintain and optimize the fidelity of education and contents and (c) *confusion bias:* it was controlled by randomizing.

## Discussion

Nursing intervention “Teaching: Individual” to increase adherence to therapeutic regimen in hypertension and / or type-2 diabetes patients represent an integral-care focus that targets low-income populations. We used a multi-component intervention comparable to research studies carried out elsewhere, where these types of intervention have been shown to improve adherence to therapeutic regimen [10, 20, 21].

Multiple examples existing in the literature have shown that educational interventions with a multi-component focus achieve adherence to the different recommendations of the health team, reduce SBP and HbA1c levels [20, 21].

This study, innovative in our milieu, will assist health authorities implementing educational interventions and formulating health policies to favor adherence to therapeutic regimen.

## Abbreviations

CARDIECOL: Conocimiento y Acción para Reducir la Dimensión de la Enfermedad Cardiovascular en Colombia (Knowledge and Action to Reduce Dimension of Cardiovascular Illness in Colombia)
